# Efficacy of ^11^C-2β-carbomethoxy-3β-(4-fluorophenyl) tropane positron emission tomography combined with ^18^F-fluorodeoxyglucose positron emission tomography in the diagnosis of early Parkinson disease: A protocol for systematic review and meta analysis

**DOI:** 10.1097/MD.0000000000023395

**Published:** 2020-12-18

**Authors:** Lei Jiang, Xixian Wang, Pengtao Li, Zhaohai Feng, Xin Shi, Hua Shao

**Affiliations:** a Department of Neurosurgery; b Department of Imaging, The First Affiliated Hospital of Xinjiang Medical University, Urumqi, Xinjiang Uygur Autonomous Region, China.

**Keywords:** ^11^C-CFT PET, ^18^F-fluorodeoxyglucose positron emission tomography, diagnosis, early Parkinson's disease, meta analysis, protocol, systematic review

## Abstract

**Background:**

Parkinson's disease (PD) has a high incidence in the elderly, and the late stage seriously affects the daily life of the patients. Most of the initial symptoms of PD are not obvious or atypical, which brings difficulties to the early diagnosis. Replacement therapy and neuroprotection after early diagnosis can significantly improve the prognosis and quality of life of patients. More and more evidence shows that ^11^C-2β-carbomethoxy-3β-(4-fluorophenyl) tropane positron emission tomography (^ 11^C-CFT PET) combined with ^18^F-fluorodeoxyglucose positron emission tomography (^18^F-FDG PET) can effectively improve the accuracy of early diagnosis. However, there is no consistent conclusion at present. The purpose of this study is to evaluate the efficacy of ^11^C-CFT PET combined with ^18^F-FDG PET in the diagnosis of early PD.

**Methods:**

We will search 7 electronic databases (PubMed, EMBASE, Web of Science, Cochrane library, PsycINFO, AMED, Scopus), ongoing trials and grey literature to collect related randomized controlled trials and will use Review Manager Software 5.2 and STATA Software 16.0 for analysis and synthesis.

**Results:**

We will integrate the existing randomized controlled trials to evaluate the value of ^11^C-CFT PET combined with ^18^F-FDG PET in the diagnosis of early PD.

**Conclusion:**

Our study may prove that ^11^C-CFT PET combined with ^18^F-FDG PET can effectively diagnose early PD.

**Registration number:**

International Prospective Register of Systematic Reviews (PROSPERO): CRD42020203442.

## 1 Introduction

Parkinson's disease (PD), which affects about 2% of people over the age of 60, is a common neurodegenerative disease.^[1]^ The pathological feature is the degeneration and loss of dopaminergic neurons in substantia nigra, resulting in an irreversible decrease in dopamine content.^[2]^ PD developed irreversibly with time, and most of them died of complications such as pneumonia and urinary tract infection after long-term bedridden.^[3]^ Most PD patients have atypical clinical symptoms in the early stage (Hoehn-Yahr I, II). When dopaminergic neurons in substantia nigra are degenerated, dopamine decreases irreversibly, and typical clinical symptoms often indicate that the patient has reached a later stage (Hoehn-Yahr III ∼ V). At this time, the damage of dopaminergic neurons is as high as 50% to 80%, even if the patient is treated in time, the prognosis and quality of life are also very poor.^[4]^ The diagnosis mainly depends on the experience of clinicians, and the symptoms, signs and the efficacy of levodopa treatment are comprehensively evaluated, but there is still a lack of diagnostic gold standard and objective examination index.^[5]^ Early diagnosis of PD is very important, because replacement therapy and neuroprotection after early diagnosis can significantly improve the prognosis and quality of life of patients, but the accuracy of early diagnosis (Hoehn-Yahr I, II) is only 53%.^[6–8]^

The pathological changes induced by PD are closely related to the changes of glucose metabolism and dopamine transporter (DAT).^[9–11]^ Many studies have confirmed that PD has its unique PD-related motor pattern (PDRP) imaging in ^18^F-fluorodeoxyglucose positron emission tomography (^18^F-FDG PET) imaging.^[12–14]^ PDRP, which is related to the decrease of dopamine, can help to detect the degeneration and loss of dopamine in substantia nigra in the early stage, and plays an important role in early diagnosis. DAT is a membrane protein in the presynaptic membrane of dopaminergic neurons, which is positively correlated with the decrease of dopaminergic neurons.^[15–19]^^11^C-2β-carbomethoxy-3β-(4-fluorophenyl) tropane positron emission tomography (^11^C-CFT PET) is an important means of DAT imaging, which can directly reflect the decrease of DAT, so that the decrease of dopaminergic neurons in early PD can be found, which is helpful for the diagnosis of early PD.^[20–23]^ However, there is no unified conclusion as to whether ^11^C-CFT PET combined with ^18^F-FDG PET can effectively diagnose early PD. The purpose of this study was to evaluate the efficacy of ^11^C-CFT PET combined with ^18^F-FDG PET in the diagnosis of early PD.

## 2 Methods and analysis

### 2.1 Study registering

We have registered the study on PROSPERO (https://www.crd.york.ac.uk/prospero/). The registration number is CRD42020203442. This study will follow the Preferred Reporting Items for Systematic Review and Meta-Analysis Protocols guidelines.^[24]^

### 2.2 Eligibility criteria

#### 2.2.1 Type of study

We will include randomized controlled trials for the diagnosis of early PD patients through ^11^C-CFT PET combined with ^18^F-FDG PET.

#### 2.2.2 Type of participants

All patients diagnosed with early PD (Hoehn-Yahr I, II) will be included in this study.

#### 2.2.3 Type of index test

Index test: early PD (Hoehn-Yahr I, II) patients were examined by ^11^C-CFT PET combined with ^18^F-FDG PET. We will exclude the combination of ^11^C-CFT PET,^18^F-FDG PET and other tests.

Reference test: early PD that meets the Parkinson's Disease Society Brain Bank (PDSBB) Parkinson diagnostic criteria will be used as the control group.^[2]^

#### 2.2.4 Outcome measurements

Outcomes are Sensitivity, Specificity, Hierarchical summary receiver operating characteristic (HSROC), Area Under the Curve (AUC), Diagnostic odds ratio (DOR), Likelihood ratio (LR).

### 2.3 Search methods for identification of studies

#### 2.3.1 Electronic data sources

From the beginning to September 25, 2020, we will search 7 electronic databases (PubMed, EMBASE, Web of Science, Cochrane library, PsycINFO, AMED, Scopus).

#### 2.3.2 Other resources

We also search for ongoing trials on the World Health Organization's International Clinical trial Registration platform. In addition, grey literature such as the health management information database, the OpenSIGLE database and the National Technical Information Service will be searched.

### 2.4 Search strategy

The search terms will be expanded around: ^11^C-CFT PET, ^18^F-FDG PET, diagnosis, early Parkinson's disease, efficacy and randomized controlled trials. There are no restrictions on race, publication date or language. For example, Table 1 shows our detailed search strategy for PubMed, and the search strategy for other databases will be adjusted according to the characteristics of each database.

**Table 1:** Search strategy used in PubMed database.

Number	Search items
1	Parkinson's/
2	(Parkinson's^∗^ or Parkinson^∗^ or Parkinsons^∗^ or Parkinsonism^∗^ or Parkinsonian^∗^ or PD^∗^).ti,ab,cl,oa,kw.
3	1 or 2
4	(^11^C-2β-carbomethoxy-3β-(4-fluorophenyl) tropane positron emission tomography^∗^ or ^11^C-CFT-PET-CT^∗^ or ^11^C-CFT-PET^∗^ or ^11^C-CFT^∗^ or C-CFT^∗^).ti,ab,cl,oa,kw.
5	3 and 4
6	(^18^F-fluorodeoxyglucose positron emission tomography^∗^ or ^18^Fluorine flurodeoxyglucose PET-CT^∗^ or ^18^F FDG PET-CT^∗^ or ^18^F-FDG-PET^∗^ or ^18^fluorodeoxy^∗^ or ^18^fluorodeoxy^∗^ or FDGPET^∗^ or ^18^F-FDG^∗^ or fluorodeoxyglucose f^18∗^).ti,ab,cl,oa,kw.
7	5 and 6
8	(randomized controlled trial^∗^ or randomized^∗^ or randomly^∗^ or RCT).ti,ab,cl,oa,kw.
9	7 and 8
10	Remove duplicates from 9

### 2.5 Data collection

#### 2.5.1 Selection of studies

**Figure 1:**
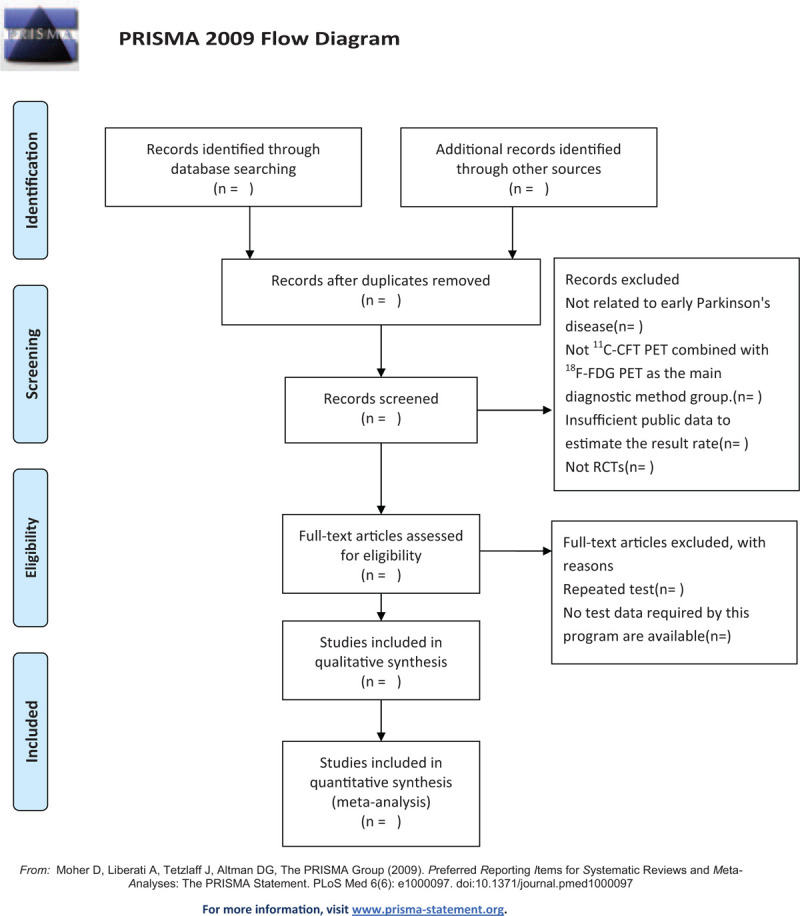
The Preferred Reporting Items for Systematic Reviews and Meta-analyses Protocols flow diagram of the study selection process.

The studies extracted from the database according to the search strategy will be imported into Endnote Software X9.0 for deduplication. Two researchers (LJ and XS) will combine inclusion and exclusion criteria by independently screening the title and abstract of each study. After that, read the full text for a second screening. Two researchers (LJ and XS) will cross-examine the included study. If disagreement arises during this period, a third researcher (HS) will participate in the discussion and decide whether to include it or not. Figure 1. The Preferred Reporting Items for Systematic Reviews and Meta-analyses Protocols flow diagram of the study selection process.

#### 2.5.2 Data extraction and management

Two researchers (XXW and PTL) will independently extract the following data from the included literature: basic information, inclusion criteria, exclusion criteria, participant information, trial and control group details, results, conclusions, and follow-up. The third researcher (ZHF) will verify the data. Contact the author of the document to obtain complete data if necessary.

### 2.6 Quality assessment

The 2 researchers (LJ and PTL) will use QUADAS-2 to assess the risk of bias in 4 areas: patient selection, indicator testing, reference criteria, and process and timing.^[27]^ If there is any dispute, the third researcher (XXW) will participate in the negotiation and settlement.

### 2.7 Statistical analysis

We will use Review Manager Software 5.2 and STATA Software 16.0 to analyze the extracted data. Summarize the specific characteristics and research results through the table. We will estimate outcome as descriptive statistics and 95% confidence intervals, and will perform plots of descriptive forest and summary receiver operating characteristic. Heterogeneity will be checked by *I*^2^ statistic. *I*^2^ ≤ 50% suggests low heterogeneity, and Mante-Haenszel fixed-effects model will be used, while *I*^2^ > 50% indicates significant heterogeneity, and Mante-Haenszel random-effects model will be applied. When the heterogeneity is low, we will conduct a meta-analysis on the basis of a sufficiently qualified study of the same result index. When there is substantial heterogeneity, we will conduct a group analysis to identify its possible sources.

### 2.8 Subgroup analysis

We will conduct a subgroup analysis according to different age of diagnosis, different symptoms and different Hoehn-Yahr grades to observe the possible heterogeneity in the study.

### 2.9 Sensitivity analysis

For studies with a risk of bias, data, and sample size deficiencies, sensitivity analyses are performed to assess robustness if statistically significant heterogeneity exists.

### 2.10 Reporting bias

We will use funnel plots and trim and fill methods to detect report biases.^[25,26]^

### 2.11 Ethics and dissemination

Considering that our study is not related to individual patient data, ethical approval is not necessary. The results of this study will be published in peer-reviewed journals or related conferences to evaluate the efficacy of ^11^C-CFT PET combined with ^18^F-FDG PET in the diagnosis of early PD.

## 3 Discussion

PD seriously affects the quality of life of the elderly, especially late PD. It is difficult to diagnose PD in the early stage, and there is still lack of diagnostic gold standard and objective examination index. More and more evidence shows that ^11^C-CFT PET combined with ^18^F-FDG PET can effectively improve the accuracy of early diagnosis, but there is no systematic review or meta analysis. This study is the first time to conduct a systematic review and meta analysis of the efficacy of ^11^C-CFT PET combined with ^18^F-FDG PET in the diagnosis of early PD. The results of this study will provide reference for clinical practice and promote follow-up research.

## Author contributions

**Conceptualization**: Lei Jiang, Xixian Wang.

**Data management**: Xixian Wang, Pengtao Li, Zhaohai Feng.

**Funding acquisition**: Xixian Wang, Lei Jiang, Hua Shao.

**Methodology**: Xin Shi.

**Software analysis**: Lei Jiang.

**Supervision**: Hua Shao.

**Writing – original draft**: Lei Jiang.
